# Relationship between living alone and common mental disorders in the 1993, 2000 and 2007 National Psychiatric Morbidity Surveys

**DOI:** 10.1371/journal.pone.0215182

**Published:** 2019-05-01

**Authors:** Louis Jacob, Josep Maria Haro, Ai Koyanagi

**Affiliations:** 1 Faculty of Medicine, University of Versailles Saint-Quentin-en-Yvelines, Montigny-le-Bretonneux, France; 2 Research and Development Unit, Parc Sanitari Sant Joan de Déu, Universitat de Barcelona, Fundació Sant Joan de Déu, Barcelona, Spain; 3 Instituto de Salud Carlos III, Centro de Investigación Biomédica en Red de Salud Mental, CIBERSAM, Madrid, Spain; Stellenbosch University, SOUTH AFRICA

## Abstract

Given the high prevalence of common mental disorders (CMDs) and individuals living alone in the United Kingdom, the goal of this study using English nationally representative data was to examine the association between living alone and CMDs, and to identify potential mediating factors of this association. The data were drawn from the 1993, 2000 and 2007 National Psychiatric Morbidity Surveys. CMDs were assessed using the Clinical Interview Schedule-Revised (CIS-R), a questionnaire focusing on past week neurotic symptoms. The presence of CMDs was defined as a CIS-R total score of 12 and above. Multivariable logistic regression and mediation analyses were conducted to analyze the association between living alone and CMDs, and to identify mediators in this association. The prevalence of CMDs was higher in individuals living alone than in those not living alone in all survey years. Multivariable analysis showed a positive association between living alone and CMDs in all survey years (1993: odds ratio [OR] = 1.69; 2000: OR = 1.63; and 2007: OR = 1.88). Overall, loneliness explained 84% of the living alone-CMD association. Living alone was positively associated with CMDs. Interventions addressing loneliness among individuals living alone may be particularly important for the mental wellbeing of this vulnerable population.

## Introduction

Globally, the lifetime prevalence of common mental disorders (CMDs) is around 30% [1]. CMDs have a major impact on quality of life [2], physical illness [3] and mortality [4]. For example, depressive disorders were the second and anxiety disorders the sixth leading causes of years lived with disability in the Global Burden of Disease 2010 study, respectively [5,6]. The effects of CMDs are not limited to the individual level, and these chronic conditions are associated with an important economic burden [7]. Therefore, there is a need for a better understanding of the risk factors of CMDs in order to improve the prevention, management and treatment of these disorders.

In the past decades, there has been a growing interest in the association between living alone and CMDs [8–18], partly driven by the fact that in many settings, the proportion of individuals living alone is increasing due to factors such as population ageing [19], lowering fertility [20], decreasing marriage rates [21], and increasing divorce rates [22]. Although most previous studies on this topic have found that living alone increases risk for CMD, they have several limitations. First, since most of these studies were conducted in the elderly, their results cannot be generalized to younger adults [12,13,15–18]. Second, the analyses often focused exclusively on depression and did not include other psychiatric conditions such as anxiety or obsessive-compulsive disorders [9,12–16,18], despite the fact that these disorders represent an important share of CMDs and are highly debilitating conditions [23–25]. Finally, to the best of our knowledge, to date, there are only a few studies which have quantified the extent to which various factors can explain the association between living arrangements and CMDs [10,12,14]. For example, factors such as obesity [26,27], smoking status [28,29], alcohol dependence [30,31], drug use [32,33], loneliness [34,35] and social support [36,37] are known to be more common in those living alone, and are risk factors for psychiatric disorders.

Therefore, our goal was to examine the association between living alone and CMDs, and to identify the factors that may be important in this association using nationally representative community-based data from the 1993, 2000 and 2007 National Psychiatric Morbidity Surveys. The UK is a particularly apposite setting to examine this association given the high prevalence of CMDs [38] and individuals living alone in this setting [39]. In particular, the magnitude of loneliness in the UK is such that a minister of loneliness has recently been appointed to tackle this problem in this country. Assessing the association between living alone and CMDs, and the factors that may underlie this association is important for the identification of vulnerable populations and the establishment of effective strategies to improve population mental health.

## Materials and methods

### Study participants

The data were drawn from the 1993, 2000 and 2007 National Psychiatric Morbidity Surveys (NPMS) [40]. Although the surveys have similar overall characteristics, there were some differences. For example, the 1993 and 2000 surveys were carried out by the Office for National Statistics in the UK (England, Wales and Scotland), while the 2007 survey was undertaken by the National Centre for Social Research in England and thus only included data from England. Furthermore, only participants aged 16–64 years and 16–74 years were included in the first and second surveys, respectively, while the third survey was conducted among all individuals aged ≥16 years. In addition, the 1993 and 2000 surveys were carried out from January to April, while the 2007 survey was carried out from January to December. Finally, the first survey was paper-based, whereas the 2000 and the 2007 were computer-based.

The multistage-stratified probability sampling design was similar across the 1993, 2000 and 2007 surveys, with the sampling frame consisting of the small user postcode address file, and the primary sampling units of postcode sectors [41]. Sampling weights were constructed to account for non-response and the probability of being selected, so that the sample was representative of the adult household population of the United Kingdom (1993 and 2000 surveys) or England (2007 survey). None of the surveys recruited previous participants or sampled from identical areas. The survey response rate was 79% for the year 1993 (n = 10108), 67% for the year 2000 (n = 8580) and 57% for the year 2007 (n = 7403). Finally, institutional review board approval was obtained and all participants provided informed consent before their inclusion.

### Dependent and independent variables

#### Common mental disorders (dependent variable)

CMDs were assessed using the Clinical Interview Schedule-Revised (CIS-R), a questionnaire focusing on past week neurotic symptoms. The presence of CMDs was defined as a CIS-R total score of 12 and above [38]. The reliability and validity of the CIS-R have been reported in previous publications [42,43].

#### Living arrangement (independent variable)

Living arrangement was assessed with the number of persons in the household, with “1” coded as “living alone” and “>1” coded as “not living alone”.

#### Control variables

The selection of the control variables was based on past literature [8–18] and included sex (male or female), age (16–34, 35–59 or 60–64 years), ethnicity (British White or other), employment status (employed, unemployed or economically inactive) and level of education: high—A-level (advanced level) or higher, implying education until the age of at least 18 years; medium—O-level (ordinary level) or GCSE (General Certificate of Secondary Education), indicating having left school at the UK statutory school, leaving age of 16 years; or low—implying having left school before the age of 16 years and/or with no educational qualifications.

#### Mediating variables

The potential mediating variables were selected based on previous studies which have shown that they are associated with living arrangement [26,29,31,33,34,36], and are risk factors for CMDs [27,28,30,32,35,37].

**Obesity.** Body mass index (BMI) was calculated as weight in kilograms divided by height in meters squared based on self-reported weight and height. Using the standard WHO definition, obesity was defined as ≥30 kg/m^2^ [44].

**Smoking status**. Current smoking was a binary variable based on the question “Do you currently smoke cigarettes at all?”, with yes and no answer options [40].

**Alcohol dependence.** Excessive alcohol consumption was screened using the Alcohol Use Disorders Identification Test (AUDIT) [45]. Alcohol dependence was assessed with the Severity of Alcohol Dependence Questionnaire (SADQ-C) in participants with an AUDIT score of 10 or above [46]. Scores of four or above indicated alcohol dependence in the past six months.

**Drug use.** Each individual was asked if he/she had used in the past year one of the following drugs: cannabis, amphetamines, cocaine, crack, ecstasy, heroin, acid or LSD, magic mushrooms, methadone or physeptone, tranquilizers, amyl nitrate, anabolic steroids, and glues [44]. Those who claimed to have used any of these drugs were considered drug users.

**Loneliness.** This was assessed with an item from the Social Functioning Questionnaire (SFQ) [47]. Respondents were asked to assess to what extent they had felt “lonely and isolated from other people” in the past two weeks with the response options, “very much”, “sometimes”, “not often” and “not at all”. In the analyses that follow, these response options were dichotomized with those who responded “sometimes” and “very much” being categorized as lonely [48].

**Social support.** This was assessed with a 7-item measure. Using answer options “not true” (score = 0), “partly true” (score = 1) and “certainly true” (score = 2), participants responded to statements which inquired if, family and friends did things to make them happy, made them feel loved, could be relied on no matter what, would see that they were taken care of no matter what, accepted them just the way they are, made them feel an important part of their lives, and gave them support and encouragement. Responses were added to create a scale score that could range from 0 to 14. The internal consistency of the scale was good: Cronbach’s α = 0.89.

### Statistical analyses

All analyses were performed with Stata version 13.1 (Stata Corp LP, College Station, Texas). The sample weighting and the complex study design were taken into account in all analyses. The level of statistical significance was set at p < 0.05.

In order to have comparable samples across surveys, the data were restricted to participants aged 16–64 years from England. Differences in the sample characteristics by living arrangement were tested using Chi-squared tests in the three separate datasets. Effect sizes were estimated as Phi for dichotomous categorical variables, and as Cramer’s V for categorical variables with more than two categories. We conducted multivariable logistic regression analysis adjusted for sex, age, ethnicity, employment status and level of education to assess the association between living alone (independent variable) and CMDs (dependent variable) separately for each survey. The logistic regression analysis was further stratified by sex and age (16–34, 35–59 and 60–64 years). Finally, using the 2007 dataset, we tested whether smoking status, alcohol dependence and drug use are effect modifiers in the association between living alone and depression by including product terms (living alone X smoking, living alone X alcohol dependence, living alone X drug use) in the fully adjusted model.

Mediation analysis was conducted to investigate the specific contribution of obesity, smoking status, alcohol dependence, drug use, loneliness, and social support in the living alone-CMD relationship. This analysis only used data from the 2007 survey because most of the variables were missing from other surveys, and included all individuals aged ≥16 years from England. We used the khb (Karlson Holm Breen) command in Stata for the mediation analysis [49]. This method can be applied in logistic regression models and decomposes the total effect (i.e., unadjusted for the mediator) of a variable into direct (i.e., the effect of living alone on CMDs adjusted for the mediator) and indirect effects (i.e., the mediational effect). Using this method, the percentage of the main association explained by the mediator can also be calculated (mediated percentage). The mediation analysis controlled for sex, age, ethnicity, employment status and level of education.

## Results

**Fig 1:**
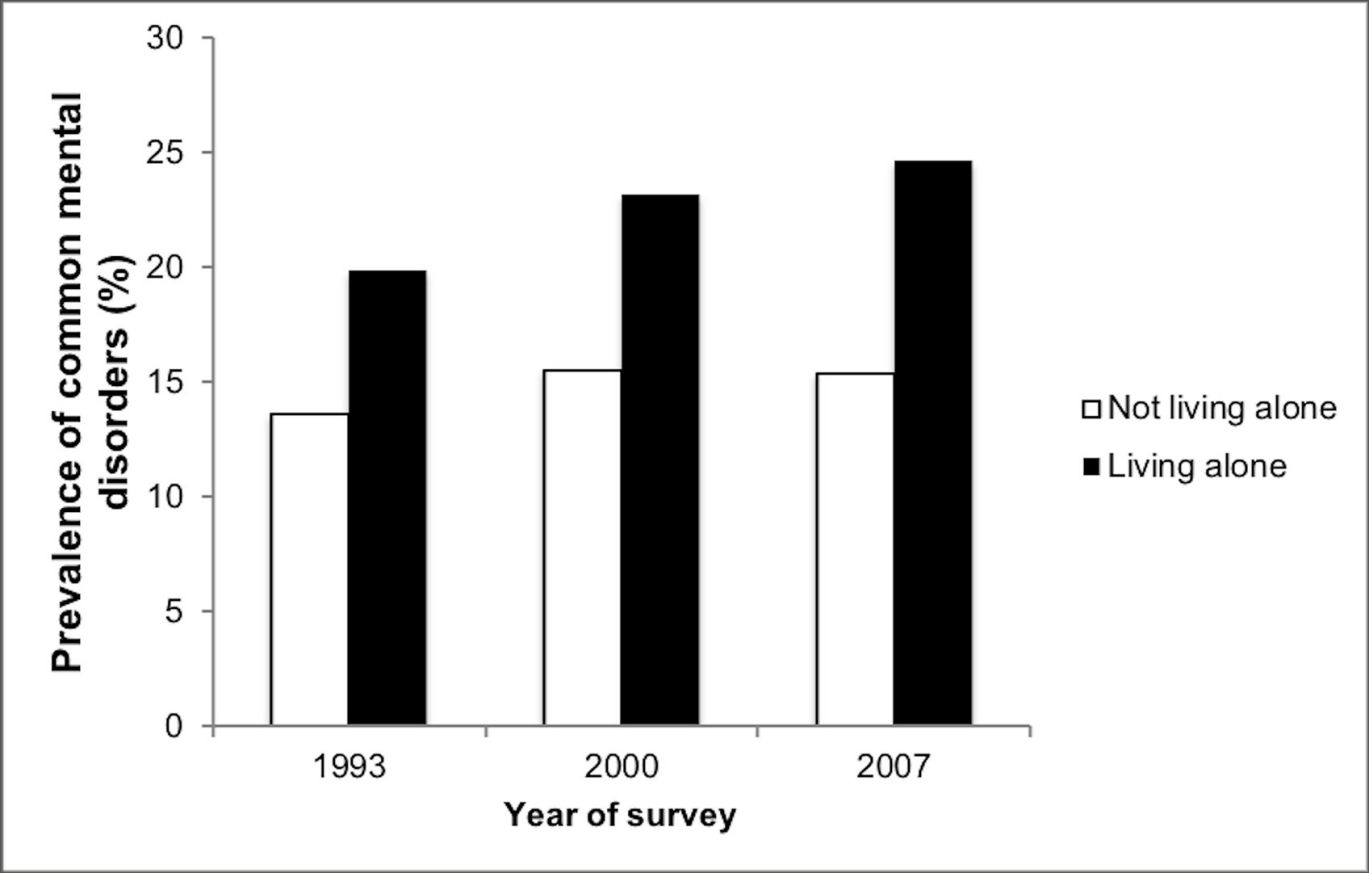
Prevalence of common mental disorders by living arrangement. Common mental disorders were assessed using the Clinical Interview Schedule-Revised (CIS-R), a questionnaire focusing on past week neurotic symptoms. The presence of common mental disorders was defined as a CIS-R total score of 12 and above.

Of the 20503 individuals from England aged 16–64 years, 8903 were from the 1993 survey, 6175 from the 2000 survey, and 5425 from the 2007 survey (**Table 1**). The prevalence of those living alone in the respective surveys were 8.8%, 9.8% and 10.7%. The corresponding figures for CMD were 14.1%, 16.3%, and 16.4%, respectively. Male sex, older age and unemployment were more frequent in participants living alone than in those not living alone, while the distribution of education was significantly different between the two groups. Details of the relationship status of the participants by year and living arrangement (alone or not) are shown in **Table 2**. The answer options for the survey conducted in 2000 were different from those of 1993 and 2007 but across all surveys, there were very few people who were married but living alone. The prevalence of CMDs was higher in individuals living alone than in those not living alone in all surveys (1993: 19.9% versus 13.6%; 2000: 23.2% versus 15.5%; and 2007: 24.7% versus 15.4%; **Fig 1**). The results of the multivariable logistic regression analysis are shown in **Table 3**. There was a positive association between living alone and CMDs in the 1993 (odds ratio [OR] = 1.69; 95% confidence interval [CI]: 1.44–2.00), 2000 (OR = 1.63; 95% CI: 1.37–1.93) and 2007 datasets (OR = 1.88; 95% CI: 1.57–2.26). This association remained significant in the stratified analyses except in people aged 60–64 years in 1993 (OR = 1.59; 95% CI: 0.99–2.55) and 2000 (OR = 1.30; 95% CI: 0.76–2.22). The non-significance among the older population may have been due to lack of statistical power as the proportion of individuals in this age group was small in the 1993 and 2000 surveys (i.e., <8%). No significant interactions were found for smoking status, alcohol dependence and drug use in the 2007 dataset. **Table 4** displays the results of the mediation analysis using data from 7403 people aged ≥16 years from England. Overall, loneliness explained 84% of the living alone-CMD association and no other significant mediators explaining more than 20% of this association were identified.

**Table 1:** Sample characteristics by year (overall and by living arrangement).

						Year of survey										
			1993 (n = 8903)					2000 (n = 6175)					2007 (n = 5425)			
			Living alone					Living alone					Living alone			
Characteristics	Category	Overall	No	Yes	Effect size^1^	p-value^2^	Overall	No	Yes	Effect size^1^	p-value^2^	Overall	No	Yes	Effect size^1^	p-value^2^
Sex	Male	50.5	49.8	57.3	0.07	<0.001	50.2	49.4	57.5	0.08	<0.001	49.7	48.7	57.6	0.07	<0.001
	Female	49.5	50.2	42.7			49.8	50.6	42.5			50.3	51.3	42.4		
Age (years)	16–34	44.3	45.0	36.9	0.08	<0.001	39.8	40.9	29.6	0.12	<0.001	37.9	38.9	29.4	0.10	<0.001
	35–59	47.9	47.8	49.0			52.4	52.0	55.8			52.9	52.4	57.4		
	60–64	7.8	7.2	14.2			7.9	7.1	14.6			9.2	8.7	13.3		
British White	No	6.8	6.9	5.6	0.01	0.138	8.1	8.0	9.1	-0.02	0.345	11.4	11.3	12.4	0.01	0.471
	Yes	93.2	93.1	94.4			91.9	92.0	90.9			88.6	88.7	87.6		
Employment	Employed	69.0	69.5	64.0	0.07	<0.001	75.0	76.1	64.7	0.09	<0.001	72.6	73.2	67.5	0.05	0.009
	Unemployed	8.5	8.0	13.1			3.2	2.9	5.3			3.5	3.5	4.2		
	Economically inactive	22.5	22.4	22.8			21.8	20.9	30.0			23.8	23.3	28.3		
Level of education	High	34.3	33.8	39.5	0.06	<0.001	39.1	38.6	44.2	0.08	<0.001	49.3	49.1	51.2	0.08	<0.001
	Medium	37.3	37.9	30.5			38.1	39.0	30.1			32.4	33.2	25.6		
	Low	28.5	28.3	30.1			22.8	22.5	25.6			18.3	17.8	23.2		

Data are column %.

^1^ Effect sizes were estimated as Phi for sex and ethnicity, and as Cramer’s V for age, employment status and level of education.

^2^ P-values were based on Chi-squared tests.

**Table 2:** Relationship status by year (overall and by living arrangement).

					Year of survey										
		1993 (n = 8903)					2000 (n = 6175)					2007 (n = 5425)			
		Living alone					Living alone					Living alone			
Category	Overall	No	Yes	Effect size^1^	p-value^2^	Overall	No	Yes	Effect size^1^	p-value^2^	Overall	No	Yes	Effect size^1^	p-value^2^
Married	59.5	65.0	2.2	0.58	<0.001	54.1	59.9	0.4	0.52	<0.001	51.4	57.5	0.6	0.63	<0.001
Cohabiting	7.2	7.9	0.2			NA^3^	NA^3^	NA^3^			12.6	14.1	0.0		
Single	24.2	21.5	52.6			32.7	30.5	52.1			27.0	23.3	57.5		
Widowed	2.1	1.1	12.7			1.8	1.0	9.2			1.6	0.7	8.9		
Divorced	5.3	3.2	26.4			8.5	6.3	28.8			5.3	3.0	24.6		
Separated	1.7	1.3	5.9			3.0	2.3	9.5			2.1	1.3	8.4		

Data are column %.

^1^ Effect sizes were estimated as Cramer’s V.

^2^ P-values were based on Chi-squared tests.

^3^ The answer option “Cohabiting” was not available for the 2000 survey.

**Table 3:** Association between living alone (independent variable) and common mental disorders (dependent variable) estimated by logistic regression.

		Year of survey					
		1993 (n = 8903)		2000 (n = 6175)		2007 (n = 5425)	
		OR	95% CI	OR	95% CI	OR	95% CI
Overall		1.69***	1.44–2.00	1.63***	1.37–1.93	1.88***	1.57–2.26
Stratified by sex	Male	1.77***	1.40–2.24	1.39*	1.08–1.79	1.87***	1.46–2.39
	Female	1.63***	1.32–2.02	1.78***	1.42–2.23	1.85***	1.45–2.35
Stratified by age	16–34 years	1.60***	1.23–2.07	1.47*	1.06–2.05	1.48*	1.03–2.14
	35–59 years	1.76***	1.43–2.17	1.70***	1.38–2.10	2.00***	1.60–2.50
	60–64 years	1.59	0.99–2.55	1.30	0.76–2.22	2.43**	1.39–4.26

Abbreviations: CI Confidence Interval; OR Odds Ratio.

The presence of common mental disorders was assessed using the Clinical Interview Schedule-Revised (CIS-R).

The overall model was adjusted for sex, age, ethnicity, employment status and level of education. The model stratified by sex was adjusted for age, ethnicity, employment status and level of education. The model stratified by age was adjusted for sex, ethnicity, employment status and level of education.

* P-value<0.05.

** P-value<0.01.

*** P-value<0.001.

**Table 4:** Mediating factors in the association between living alone and common mental disorders (2007 dataset).

	Total effect		Direct effect		Indirect effect		
Mediator	OR [95%CI]	P-value	OR [95%CI]	P-value	OR [95%CI]	P-value	%Mediated
Obesity	1.59 [1.36,1.87]	<0.001	1.62 [1.38,1.90]	<0.001	0.99 [0.97,1.00]	0.009	NA^1^
Smoking status	1.63 [1.40,1.91]	<0.001	1.57 [1.34,1.84]	<0.001	1.04 [1.02,1.06]	<0.001	8
Alcohol dependence	1.65 [1.41,1.93]	<0.001	1.61 [1.38,1.89]	<0.001	1.02 [1.01,1.04]	0.008	4
Drug use	1.62 [1.39,1.90]	<0.001	1.57 [1.34,1.83]	<0.001	1.04 [1.02,1.06]	<0.001	7
Loneliness	1.54 [1.29,1.83]	<0.001	1.07 [0.90,1.28]	0.449	1.43 [1.35,1.52]	<0.001	84
Social support	1.61 [1.37,1.89]	<0.001	1.49 [1.27,1.75]	<0.001	1.08 [1.05,1.11]	<0.001	17

Abbreviations: CI Confidence Interval; OR Odds Ratio.

The presence of common mental disorders was assessed using the Clinical Interview Schedule-Revised (CIS-R).

Model was adjusted for sex, age, ethnicity, employment status and level of education.

^1^ Mediated percentage was only calculated when indirect effect was positive and significant (P<0.05).

## Discussion

### Main findings

This study using nationally representative community-based data of more than 20500 individuals showed that the prevalence of CMDs was higher in people living alone than in those not living alone. Furthermore, the results of the multivariable logistic regression analysis showed that people living alone had a significant 1.39–2.43 times higher odds for CMDs. This association was observed in all age groups including young adults and both sexes, and the magnitude of the association remained relatively stable between 1993 and 2007. Finally, overall, the relationship between living alone and CMDs was largely mediated by loneliness.

### Interpretation of the findings

Several authors have investigated the association between living alone and CMDs [8–18]. In the late 1990s, researchers from the UK found that living alone was associated with a 1.3-fold increase in the risk of diagnosis of depression and anxiety [8]. The main limitation of this study is that it focused on sociodemographic factors and did not account for other variables such as behavioral factors. Later, in 2008, another study conducted in the same country showed that the prescription of antidepressant, anxiolytic and hypnotic drugs was higher in people living alone than in those not living alone [11]. Similar findings have been reported from other settings. For example, in a population-based sample of 4685 adults from Finland, there was a 2-fold increase in the risk of having anxiety or depressive disorders in people living alone compared to those who were married [10]. Therefore, our findings are in line with existing literature but extend previous knowledge by showing that the association between living alone and CMDs has remained relatively stable between 1993 and 2007, and that this association is ubiquitous regardless of age and sex.

Based on data from 2007, we found that loneliness was the strongest significant explanatory factor of the association between living alone and CMDs, explaining approximately 84% of the association overall. Loneliness has previously been reported to be more frequent in people living alone than in those living with a spouse [34]. In our study, the prevalence of loneliness among those living alone in 2007 was 34% and this figure was much higher than in those who were not living alone (18%). Loneliness has been prospectively associated with the subsequent emergence of psychiatric disorders such as depression and anxiety [50,51]. It has been suggested that loneliness may lead to CMDs via rumination [52], negative appraisals of social company [53], immune dysregulation [54] or addiction [55]. Regarding rumination, previous research conducted in the US revealed that rumination mediates the association between loneliness and depressed mood in college students, suggesting that negative cognitive and emotional self-regulatory strategies have a major impact on mental health [56]. Negative appraisals of social company also play a major role in the association between loneliness and CMDs, and this involves hostility [57] and social phobia [58]. Finally, researchers have previously observed that perceived social isolation increases the risk of immune dysregulations [59], and both immune suppression and activation are known to be key features of depression and other mental disorders [60].

### Clinical implications and directions for future research

Our study results indicate that living alone may be a risk factor for CMDs regardless of age and sex and that loneliness may be an important mediating factor. Clinicians should be aware that those living alone have a higher prevalence of CMDs and that this may largely be explained by loneliness. Previously reported interventions to decrease levels of loneliness include social facilitation interventions, psychological therapies, health and social care provision, animal interventions, befriending interventions and leisure/skill development [61]. Given that our study was of cross-sectional design, future studies of longitudinal design are warranted to provide more concrete evidence regarding causality and temporal associations. Future studies should also seek to identify other potential mediators such as cortisol dysregulations [62,63] or changes in brain structure [64,65] that were not assessed in our study. Investigating cortisol dysregulations is important because it has been found that people living alone are at a particular risk for elevated night time cortisol and flat diurnal slope [63], and that cortisol is a key player in the development of major depressive disorder [62]. Social exclusion and loneliness are also associated with major changes in the brain (e.g., decreased activity of cortical regions involved in mentalizing) [65], while imaging studies have reported changes in the volume of several cerebral regions (e.g., hippocampus, amygdala) in CMDs [64]. Finally, given that cognition (especially social cognition) is frequently impaired in individuals with low levels of social interaction [66] and people with mental disorders [67], future studies should assess its mediating and moderating role in the relationship between independent living and CMDs.

### Strengths and limitations

The large sample size and the use of three nationally representative surveys are the main strengths of the study. Nonetheless, there are several limitations that should be mentioned. First, the declining response rates in the successive surveys are problematic and may have introduced bias in our results. However, previous research has highlighted the fact that the risk estimates in the identification of risk factors of psychiatric conditions are only minimally affected by low response rates [68,69]. Second, since the analysis did not include the homeless, the present results cannot be generalized to this population, where living alone is very common. Third, as self-reports were used to assess CMDs, and as CMDs are frequently associated with stigma [70], it is possible that we have underestimated the prevalence of these disorders. Finally, since the design of this work was cross-sectional, causality or temporality in the relationship between living alone and CMDs cannot be determined. Thus, future longitudinal studies are warranted.

### Conclusion

Living alone was positively associated with CMDs regardless of sex or age, and this association was largely explained by loneliness. Based on these findings, prevention of CMDs in people living alone should consider all ages and targeting loneliness in particular may be important.
